# Could Pulsed Wave Tissue Doppler Imaging Solve the Diagnostic Dilemma of Right Atrial Masses and Pseudomasses? A Case Series and Literature Review

**DOI:** 10.3390/jcm14010086

**Published:** 2024-12-27

**Authors:** Andrea Sonaglioni, Gian Luigi Nicolosi, Giovanna Elsa Ute Muti-Schünemann, Michele Lombardo, Paola Muti

**Affiliations:** 1 Division of Cardiology, IRCCS MultiMedica, 20123 Milan, Italy; michele.lombardo@multimedica.it; 2 Division of Cardiology, Policlinico San Giorgio, 33170 Pordenone, Italy; gianluigi.nicolosi@gmail.com; 3 Department of Emergency, Fondazione IRCSS Ca’ Granda, Ospedale Maggiore Policlinico, 20122 Milan, Italy; giovanna.muti@unimi.it; 4 Department of Biomedical, Surgical and Dental Sciences, University of Milan, 20122 Milan, Italy; pmuti26@gmail.com; 5 IRCCS MultiMedica, Via Fantoli 16/15, 20138 Milan, Italy

**Keywords:** right atrial masses, pseudomasses, differential diagnosis, pulsed wave tissue Doppler imaging, mass mobility assessment

## Abstract

Even if rarely detected, right atrial (RA) masses represent a diagnostic challenge due to their heterogeneous presentation. Para-physiological RA structures, such as a prominent Eustachian valve, Chiari’s network, and lipomatous atrial hypertrophy, may easily be misinterpreted as pathological RA masses, including thrombi, myxomas, and vegetations. Each pathological mass should always be correlated with adequate clinical, anamnestic, and laboratory data. However, the differential diagnosis between pathological RA masses may be challenging due to common constitutional symptoms, as in the case of vegetations and myxoma, which present with fever and analogous complications such as systemic embolism. The implementation of transthoracic echocardiography (TTE) with pulsed wave (PW) tissue Doppler imaging (TDI) may improve the visualization and differentiation of intracardiac masses through different color coding of the pathological structure compared to surrounding tissue. More remarkably, PW-TDI can provide a detailed assessment of the specific pattern of motion of each intracardiac mass, with important clinical implications. Specifically, a TDI-derived pattern of incoherent motion is typical of right-sided thrombi, myxomas, and vegetations, whereas right-sided pseudomasses are generally associated with a TDI pattern of concordant motion synchronous with the cardiac cycle. An increased TDI-derived mass peak antegrade velocity may represent an innovative marker of the embolic potential of mobile right-sided pathological masses. During the last two decades, only a few authors have used TTE implemented with PW-TDI for the characterization of intra-cardiac masses’ morphology and mobility. Herein, we report two clinical cases of totally different right-sided cardiac masses diagnosed using a multimodality imaging approach, including PW-TDI, followed at our institution. The prevalence and physiopathological characteristics of the most relevant RA masses and pseudomasses encountered in clinical practice are described in the present narrative review. In addition, we will discuss the principal clinical applications of PW-TDI and its potential value in improving the differential diagnosis of pathological and para-physiological right-sided cardiac masses.

## 1. Introduction

Right atrial (RA) masses are rarely detected in clinical practice [1]. These comprise a broad set of neoplastic and non-neoplastic lesions with significant heterogeneity in their physiopathological characteristics and clinical presentations [2]. The neoplastic RA masses may be benign, such as myxomas, papillary fibroelastomas, lipomas, and rhabdomyomas, or malignant, such as angiosarcomas, lymphomas, and metastases arising from lung, breast, melanoma, or hematologic malignancies [3]. The most common non-neoplastic RA lesions are thrombi, causing a large percentage of pulmonary emboli [4,5], and infective vegetations, commonly involving the tricuspid valve, often seen in patients with medical devices or intravenous drug abusers [6,7,8]. The correct diagnosis of RA masses is complicated due to the frequent presence of RA pseudomasses, which represent normal or para-physiological structures mimicking pathological lesions. The most frequently detected RA pseudomasses are a prominent eustachian valve [9], Chiari’s network [10], lipomatous hypertrophy of the interatrial septum [11], or lipomatous hypertrophy involving the free wall of the RA [12].

Non-invasive imaging plays a central role in the diagnosis of RA masses [13]. Despite the advantages in tissue characterization and distinction of surrounding structures of both cardiac magnetic resonance [14] and contrast-enhanced cardiac computed tomography (CT) [15], transthoracic echocardiography (TTE) still remains the first-line imaging modality for cardiac mass assessment. This is due to its wide availability, non-invasiveness, absence of contrast material or radiation exposure, and capability to achieve dynamic assessment of lesions in relation to the adjacent chambers and valves [16,17]. TTE may rapidly assess the location, size, shape, and attachment of mobile intra-cardiac masses, as well as the presence of hemodynamic consequences. Due to its higher spatial and temporal resolution, transesophageal echocardiography (TEE) may further allow better visualization and identification of small masses (<5 mm) [18]. However, TEE is limited by its invasive nature and the risk of false diagnoses resulting from misinterpretation of normal and abnormal anatomy [19]. Both TTE and TEE allow for the visualization of mobile intra-cardiac masses; however, they may not accurately assess differences in motion between anomalous and regular intra-cardiac structures. Conversely, pulsed wave (PW) tissue Doppler imaging (TDI) allows us to characterize the specific motion pattern of both pathological and para-physiological intracardiac masses [20]. Notably, PW-TDI may significantly improve the visual assessment of these structures [20] and may also provide a precise definition of mass mobility by measuring the mass peak antegrade velocity [21]. To obtain the PW-TDI spectral curve corresponding to intracardiac masses, it is necessary to place the sample volume (usually 5 mm axial size) of PW-TDI on the mobile portion of each mobile mass. To date, only a few authors have used TTE implemented with PW-TDI for characterizing intra-cardiac masses morphology and mobility [20,21,22,23,24]. In this paper, we will outline two clinical cases of RA masses diagnosed using a multimodality imaging approach, including PW-TDI, followed at our institution. Moreover, we will discuss the principal clinical applications of PW-TDI and its potential value in improving the differential diagnosis between pathological RA masses and pseudomasses.

## 2. Case Series

### 2.1. Clinical Case 1

**Figure 1:**
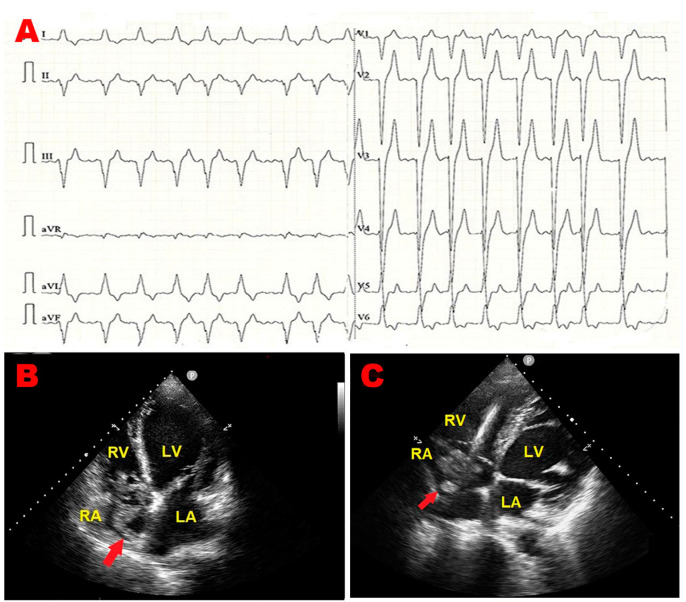

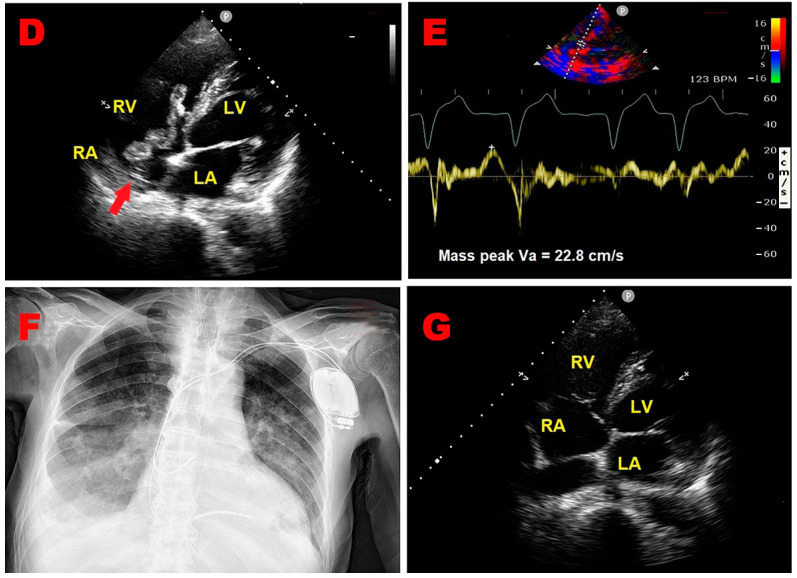
(**A**) Twelve-lead electrocardiogram, showing atrial fibrillation with left bundle branch block and rapid ventricular rate. (**B**) Transthoracic echocardiography. Apical four-chamber view, demonstrating a large, S-shaped thrombotic mass (red arrow) occupying the whole right atrial cavity. (**C**) Transthoracic echocardiography. Right ventricular focused apical four-chamber view showing the right atrial thrombotic mass (red arrow) between the Chiari network and the right ventricular lead. (**D**) Transthoracic echocardiography. Right ventricular focused apical four-chamber view showing the S-shaped right atrial thrombus tethered to Chiari’s network (red arrow), free-floating and prolapsing through the tricuspid valve into the right ventricle. (**E**) Pulsed wave tissue Doppler imaging is used to assess the right atrial mass mobility. Its motion was very rapid and uncoordinated, with increased peak antegrade velocity, measured by positioning the sample volume of pulsed wave tissue Doppler imaging on the free mobile portion of the mass. (**F**) Chest X-rays. Posteroanterior view showing hilar congestion, multifocal pneumonia, and right pleural effusion. (**G**) Transthoracic echocardiography. Right ventricular focused apical four-chamber view, revealing the complete disappearance of right atrial thrombotic mass. LA, left atrium; LV, left ventricle; RA, right atrium; RV, right ventricle; Va, antegrade velocity.

An 84-year-old male (BSA 1.63 m^2^, BMI 19 Kg/m^2^) was admitted to the emergency department of our institution due to fever, asthenia, and cough. As per past medical history, the patient was affected by chronic renal failure and ischemic dilated cardiomyopathy with reduced left ventricular ejection fraction (LVEF). He was previously implanted with a bicameral cardioverter–defibrillator. Upon physical examination, blood pressure was 126/79 mmHg, and heart rate was 123 b.p.m., with a body temperature of 38 °C. Arterial blood gas analysis revealed an oxygen saturation of 91%, pH of 7.43, mild hypoxemia (PaO_2_ = 76 mmHg), and normocapnia (PaCO_2_ = 35 mmHg). The electrocardiogram (ECG) detected atrial fibrillation (AFib) with left bundle branch block with rapid ventricular rate (Figure 1A). Blood tests showed neutrophilic leukocytosis [white blood cell count 43 × 10^9^/L (range 4–11 × 10^9^/L)], C-reactive protein of 22 mg/dL (range 0.05–0.50 mg/dL), and an estimated glomerular filtration rate of 18.5 mL/min/m^2^ with markedly elevated levels of D-dimer (>20,000 ng/mL) and N-terminal pro-B-type natriuretic peptide (NT-proBNP) (>10,000 pg/mL). An urgent TTE highlighted a large, S-shaped, and extremely mobile RA mass tethered to Chiari’s network, free-floating and prolapsing through the tricuspid valve into the right ventricle (Figure 1B–D). By placing a 5 mm sample volume of PW-TDI at the level of the mobile portion of the RA mass, a peak antegrade longitudinal velocity of 22.8 cm/s was recorded (Figure 1E). On PW-TDI, the RA mass showed a pattern of incoherent motion, with different velocities and directions compared to the surrounding myocardial tissue. Given the severe renal failure, contrast-enhanced chest CT could not be performed. Chest X-ray showed hilar congestion, multifocal pneumonia, and right pleural effusion (Figure 1F). The patient was hospitalized in the internal medicine ward. He was in poor general condition. Blood cultures and transesophageal echocardiography were not performed, and the patient was conservatively treated with anticoagulants (subcutaneous calcium–heparin 5000 I.U. every 12 h), intravenous (IV) antibiotics (piperacillin plus tazobactam 2.25 g every 8 h), IV diuretics (furosemide 40 mg/die), and beta blockers (metoprolol 150 mg/die). A follow-up TTE was performed after one week, demonstrating the complete resolution of the RA mass (Figure 1G). Accordingly, infective endocarditis was excluded, and the RA mass was judged to be compatible with a thrombus of the RA cavity entrapped within the Chiari network, which was promptly resolved through the use of antithrombotics. Despite the intense cardio-protective treatment, the patient’s condition quickly worsened, resulting in septic shock and death after two weeks of hospitalization.

### 2.2. Clinical Case 2

**Figure 2:**
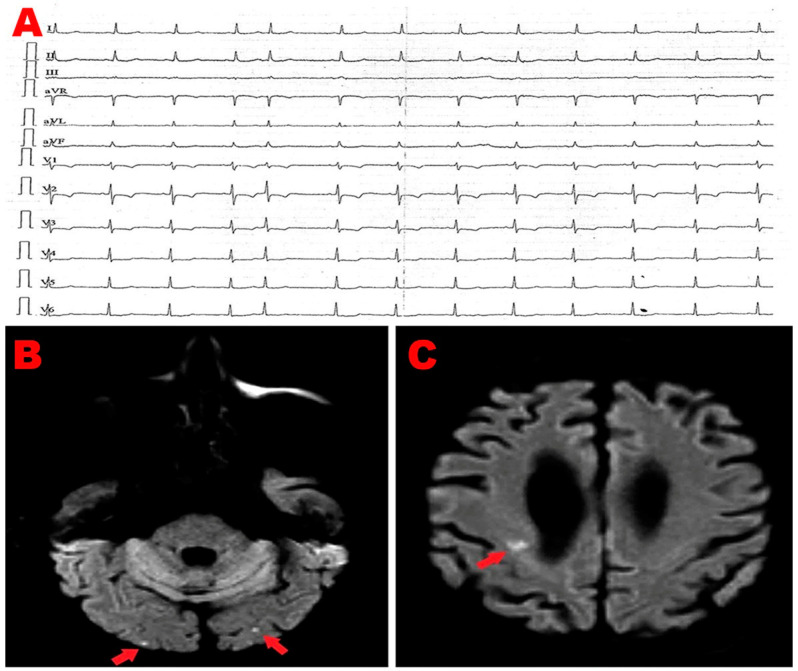

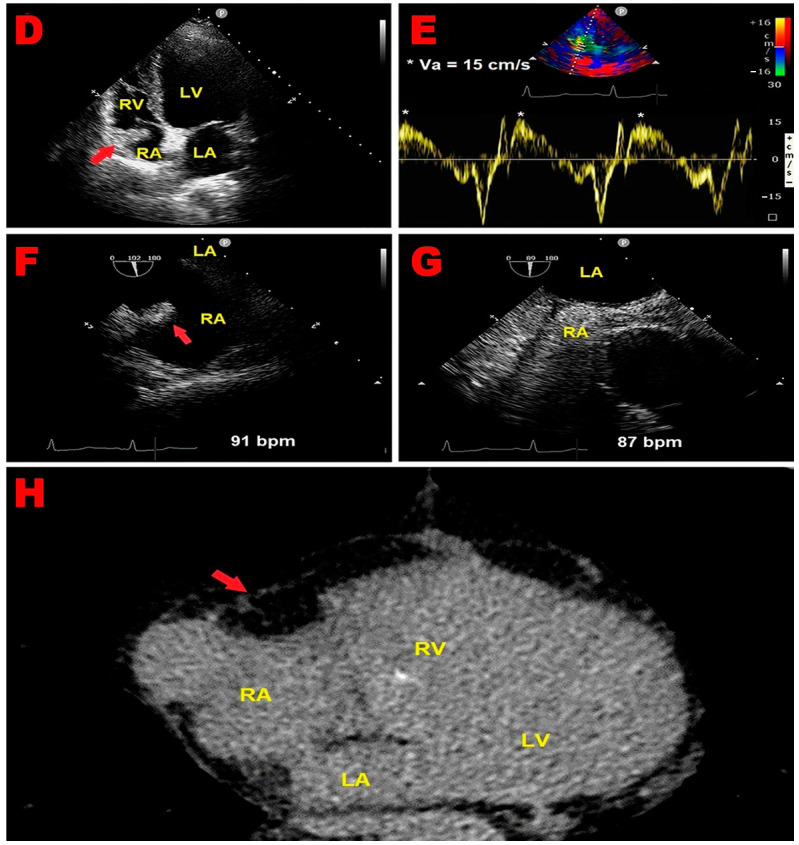
(**A**) Twelve-lead electrocardiogram, showing sinus rhythm with normal atrioventricular and intra-ventricular conduction, single supraventricular extrasystole. (**B**,**C**) Diffusion-weighted magnetic resonance imaging of the brain, revealing bilateral cortical and subcortical ischemic lesions (red arrows) involving frontal and occipital areas of both cerebral hemispheres. (**D**) Transthoracic echocardiography. Apical four-chamber view, showing a suspected RA mass (red arrow), with similar echogenicity as the myocardium, occupying the infero-lateral portion of the right atrial cavity. (**E**) PW-TDI performed to assess the mass motility. By placing a 5 mm sample volume at the level of the mobile portion of the suspected RA mass, this structure showed a cyclic motion that was concordant with surrounding myocardial tissue. The peak antegrade velocity of RA mass was 15 cm/s and remained stable at each cardiac cycle. (**F**) Transesophageal echocardiography. Mid-esophageal bicaval view, demonstrating an echogenic structure (red arrow) projecting into the RA cavity, visualized in proximity of the atrioventricular junction, in close proximity with RA infero-lateral wall. (**G**) Contrast-enhanced transesophageal echocardiography highlighting the integrity of the interatrial septum, thus excluding patent foramen ovale. (**H**) Contrast-enhanced chest CT scan showing a homogenously hypodense formation (red arrow) occupying the infero-lateral portion of RA cavity, compatible with the adipose tissue of the right atrioventricular groove. CT, computed tomography; LA, left atrium; LV, left ventricle; PW, pulsed wave; RA, right atrium; RV, right ventricle; TDI, tissue Doppler imaging; * Va, mass peak antegrade velocity.

An 83-year-old male (BSA 1.75 m^2^, BMI 22.5 Kg/m^2^) affected by chronic renal failure (estimated glomerular filtration rate: 26 mL/min/m^2^), without previous cardiovascular history, was admitted to the emergency department of our institution due to the sudden onset of symmetric weakness with paresthesias involving both legs. Relevant upon physical examination was a blood pressure of 110/70 mmHg and a stage 2 sacral decubitus ulcer. The ECG showed sinus rhythm around 60 b.p.m with normal atrioventricular and intra-ventricular conduction; a single supraventricular extrasystole was recorded (Figure 2A). The patient was admitted to the internal medicine ward, where he received IV antibiotics (ceftriaxone 2 gr/die), fluid therapy (physiological solution 1500 mL/die), and anticoagulants (subcutaneous calcium–heparin 5000 I.U. every 12 h). An electromyography identified axonal sensorimotor polyneuropathy, and the patient was diagnosed with Guillain–Barré syndrome. Upon diagnosis, the patient was transferred to the Department of Neuromotor Rehabilitation, where he underwent treatment with acetyl-L-carnitine and motor rehabilitation. During hospitalization, the patient manifested a sudden onset of aphasia and confusional state. Contrast-enhanced CT scan of the brain excluded ischemic lesions, whereas brain magnetic resonance imaging (MRI) with contrast showed bilateral cortical and subcortical ischemic lesions involving the frontal and occipital lobes of both cerebral hemispheres (Figure 2B,C). The patient underwent serial ECGs and a 24 h ECG Holter monitoring; in the short-term monitoring, there was no evidence of AFib. Carotid ultrasonography revealed bilateral mild stenosis (25% degree) of the carotid bifurcation due to deposition of calcific atherosclerotic plaques. We performed a bedside TTE, which showed small chamber sizes, normal biventricular systolic function, first-degree diastolic dysfunction, absence of relevant valvulopathies, and normal hemodynamics. From the apical four-chamber view, a suspected RA mass was detected with similar echogenicity as the surrounding myocardium. The mass occupied the infero-lateral portion of right atrial cavity, as demonstrated in Figure 2D. By placing a 5 mm sample volume of PW-TDI at the level of the mobile portion of the RA mass, this structure showed a cyclic motion concordant with the surrounding myocardial tissue. The peak antegrade velocity of RA mass was 15 cm/s and remained stable at each cardiac cycle (Figure 2E). A subsequent TEE was performed for a more detailed evaluation of the suspected RA mass and to determine whether there were other potential cardiac sources of emboli. TEE documented the same echogenic structure projecting into the RA cavity, visualized in proximity of the atrioventricular junction in close proximity of the RA infero-lateral wall (Figure 2F). Contrast-enhanced TEE demonstrated the integrity of the interatrial septum, thus excluding a patent foramen ovale (Figure 2G). We also excluded the presence of thrombi within the left atrial appendage and atherosclerotic debris. Lastly, the patient underwent contrast-enhanced chest CT scan, showing a homogenously hypodense formation occupying the infero-lateral portion of RA cavity, compatible with adipose tissue of the right atrioventricular groove (Figure 2H). In light of the aforementioned findings, the suspected RA mass was more properly defined as RA pseudomass, ascribed to the systolic infolding of the lipomatous right atrioventricular junction, in close continuity with RA infero-lateral wall. The ischemic lesions detected on brain MRI were, therefore, attributed to the cerebral embolization of carotid atherosclerosis. After three months of hospitalization, the patient was discharged on oral anticoagulation (warfarin 5 mg according to the International Normalized Ratio) and statin therapy (rosuvastatin 5 mg/die).

## 3. Discussion

RA masses are a diagnostic dilemma for clinical cardiologists. The differential diagnosis between pathological and para-physiological RA masses is complex. The most relevant RA pathological masses include thrombi, myxomas, and vegetations. Each pathological mass should be adequately correlated with clinical, anamnestic, and laboratory data. However, pathological RA masses, such as vegetations and myxomas, may share common constitutional symptoms, such as fever, and clinical complications, such as systemic embolism. The implementation of TTE with PW-TDI may improve the definition of intracardiac masses through different color coding of the pathological structure compared to the surrounding tissue. More remarkably, PW-TDI can also provide a detailed assessment of the specific pattern of motion of each intracardiac mass with important clinical implications.

Herein, we describe the most common RA masses and pseudomasses that can be detected through cardiac ultrasound in clinical practice.

### 3.1. Right Atrial Masses

#### 3.1.1. Right Atrial Thrombi

RA thrombosis is a rare finding. It may represent a complication of AFib, with about 12% of AFib-related thrombi located in the RA/right atrial appendage [25]. In the context of AFib, the incidence of RA thrombi is significantly lower compared to left atrial appendage thrombi, likely correlated to the larger width of the RAA neck and smaller RA areas in comparison to LAA and left atrial ones, thus avoiding blood stasis and subsequent clot formation [26]. RA thrombosis may occasionally be detected in patients with mechanical tricuspid valves, right-sided pacemaker leads, atrial septal closure devices, and indwelling central venous lines [27,28]. Moreover, RA thrombosis has been reported in about 10% of patients with pulmonary thromboembolism (PTE) [29]. PTE patients diagnosed with RA thrombosis have an estimated mortality rate of about 28% in treated patients and reach 80–100% in untreated cases [30]. RA thrombotic formations associated with deep vein thrombosis and pulmonary emboli are generally serpiginous, worm-like in appearance, and typically exhibit an increased mobility within the right-sided chambers [5,31]. PW-TDI may aid in distinguishing between RA thrombotic masses and the surrounding myocardial tissue. Importantly, PW-TDI may also allow us to precisely assess the thrombotic mass motility by measuring its peak antegrade velocity. Highly mobile RA thrombi are characterized by a PW-TDI pattern of incoherent motion. In the case of suboptimal echocardiographic windows, TEE may provide a better evaluation of the RA thrombus location and morphological characteristics.

#### 3.1.2. Right Atrial Myxomas

Cardiac myxoma is the most common type of benign cardiac neoplasm, with a prevalence of 50% of all benign cardiac tumors [32]. Of these neoplasms, 25% are RA myxomas occurring predominantly in females between the third and sixth decade of life [33,34,35]. These patients are usually diagnosed late due to the absence of symptoms for long periods of time. When symptomatic, they usually manifest with specific findings such as fever, weight loss, arthralgia, anemia, and embolic complications [24,36]. In addition, RA myxoma can potentially obstruct the tricuspid valve, leading to signs and symptoms of right-sided heart failure, peripheral edema, and ascites [37]. On TTE, RA myxomas may be misinterpreted as RA thrombotic formations; however, differently from RA thrombi, RA myxomas tend to be larger with a broader base, appear relatively fixed with smooth edges, and present as solid masses [38]. TTE plays a crucial role in assessing the size, location, and relationship of the mass with surrounding tissues. PW-TDI assessment may facilitate the diagnostic recognition of RA myxomas by clearly identifying their incoherent and uncoordinated motion. The mass peak antegrade longitudinal velocity, measured by placing the 5 mm sample volume at the level of the mobile portion of the RA myxoma, may represent a potential echocardiographic marker for the prediction of embolic risk [24].

#### 3.1.3. Right Atrial Vegetations

Right-sided infective endocarditis (IE) accounts for 5% to 10% of all cases of IE [39]. Its development is associated with intravenous drug use, intracardiac devices, and central venous catheters, all of which have become more prevalent over the last two decades [40]. The most common complications of right-sided IE include tricuspid insufficiency, abscess formation, and septic pulmonary embolism [41,42]. TTE is the primary diagnostic tool used for detecting right-sided vegetations. Due to the anterior location of right-sided structures, TTE may provide useful information concerning right-sided IE. However, TTE has a limited sensitivity for identifying vegetations attached to pacemaker leads [43]. In addition, lead aggregations from thrombi, which are found in up to 20% of pacemaker patients without infection, may be misinterpreted as vegetations [44]. Additionally, several pathological formations, such as RA thrombi and old noninfected vegetations, or physiological structures, such as the crista terminalis, may cause diagnostic confusion. Clinical parameters, particularly increased infectious biomarkers and the presence of fever, may aid in the differential diagnosis between right-sided vegetations and the aforementioned structures. Moreover, the implementation of TTE with PW-TDI may allow us to identify the typical pattern of incoherent motion caused by the free oscillation of vegetations. This pattern of motion is characterized by a completely different velocity and direction of motion of the pathological mass compared to the surrounding myocardial tissue. This vegetation mobility is totally disjointed from the cardiac cycle. Due to its superior sensitivity in comparison to TTE [45], TEE is mandatory in patients with suspected cardiac-device-related IE or in patients with suboptimal transthoracic imaging.

**Figure 3:**
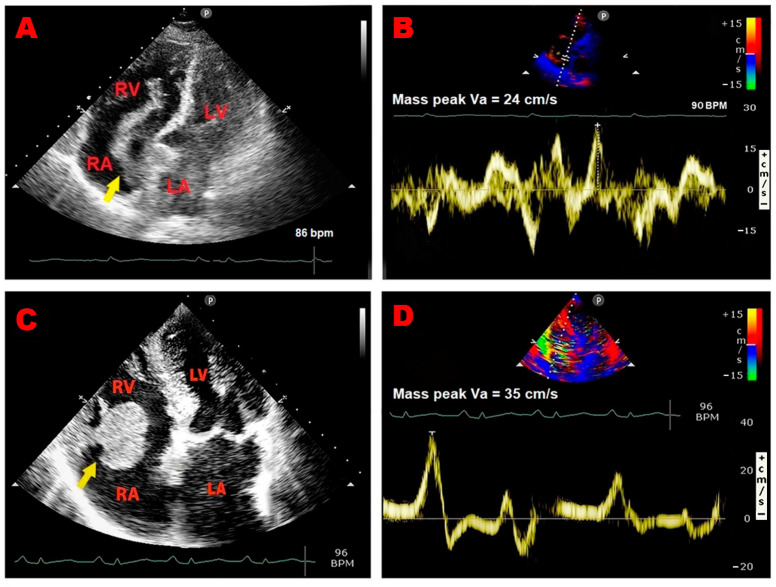

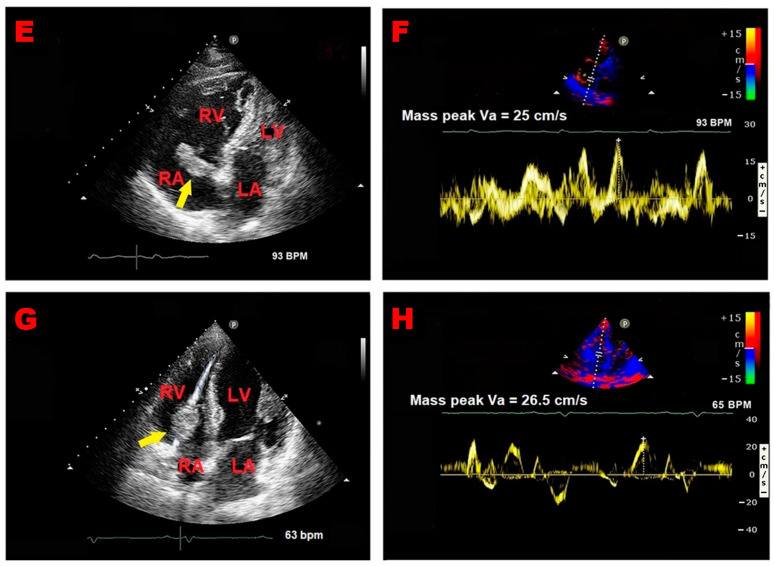
Representative examples of RA thrombus, myxoma, and vegetation assessed by TTE implemented with PW-TDI. (**A**) Transthoracic echocardiography. Apical four-chamber view, showing large S-shaped RA thrombus (yellow arrow) entrapped in the Chiari network, prolapsing through the tricuspid valve into the right ventricle. (**B**) PW-TDI assessment of the thrombotic mass motility: the pattern of incoherent motion is typical of a pathological RA mass. (**C**) Transthoracic echocardiography. Apical four-chamber view, revealing RA atrial multilobulated, hypermobile, echogenic cauliflower mass attached to the tricuspid lateral annulus with a short stalk (yellow arrow), compatible with a pedunculated myxoma. (**D**) PW-TDI assessment of the RA myxoma motility: the mass motility is totally independent of the cardiac cycle. (**E**) Transthoracic echocardiography. Apical four-chamber view, demonstrating an echogenic mass attached to the fossa ovalis, extending into the RA (yellow arrow), compatible with RA myxoma. (**F**) Pattern of uncoordinated motion of RA myxoma assessed by PW-TDI. (**G**) Transthoracic echocardiography. Apical four-chamber view, highlighting large vegetation attached to the pacemaker lead in the right atrium (yellow arrow) of a patient with infective endocarditis. (**H**) Pattern of incoherent motion of RA vegetation on PW-TDI. LA, left atrium; LV, left ventricle; PW, pulsed wave; RA, right atrium; RV, right ventricle; TTE, transthoracic echocardiography; TDI, tissue Doppler imaging; Va, antegrade velocity. (**C**,**D**) are reproduced from the paper [24] (license number 5917070978039).

Representative examples of RA thrombus, myxoma, and vegetation assessed by TTE implemented with PW-TDI are illustrated in Figure 3A–F.

### 3.2. Right Atrial Pseudomasses

#### 3.2.1. Prominent Eustachian Valve

The Eustachian valve (EV) is an embryologic remnant of the right sinus venosus valve located at the junction between the inferior vena cava (IVC) and RA. During fetal life, the EV directs incoming oxygenated blood from IVC toward the foramen ovale (FO) and away from the tricuspid valve. After the FO closure, it loses its function, and its regression during childhood may be complete or incomplete. If its regression is incomplete, the EV may persist as a mobile flap with variable thickness, length, and shape [46]. When prominent, the EV may be confused with pathological RA masses, such as vegetations, thrombi, or cor triatriatum dexter [46,47]. It is generally a benign condition. However, a prominent EV has been associated with an increased risk of paradoxical embolization (cerebral embolism) due to right-to-left shunt [48,49], especially in patients with large patent foramen ovale (PFO) or atrial septal defects without pulmonary hypertension or right ventricular outflow obstruction [50,51,52]. Indeed, the EV may contribute to maintaining an embryonic RA flow pattern into adult life and direct the blood from the IVC via PFO into the left atrium. Other potential complications associated with a large EV include IE, thrombosis, and possibly subsequent pulmonary embolism [53,54]. Occasionally, a big EV can obstruct the flow from the IVC to the right atrium. Importantly, the EV can also cause intermittent obstruction of the venous cannula inflow during its direct insertion via a median sternotomy or femoral cannulation during cardiopulmonary bypass [55].

#### 3.2.2. Chiari’s Network

The Chiari network (CN) is a fenestrated membrane located in the RA between the valve of the IVC and the valve of the coronary sinus (Thebesian valve). Its presence results from the incomplete resorption of the right sinus venosus [56]. During fetal life, the CN has the role of directing blood from the IVC toward the FO. Its prevalence ranges between 2 and 13.6% of adults [57,58]. Some authors have associated the CN with an increased risk of paradoxical embolism through right-to-left shunting [59], IE [60], or supraventricular tachyarrhythmias [61]. On the other hand, the CN may also play a protective role against the thrombo-embolic risk by holding thrombo-emboli originating from the leg or pelvic veins in the network and preventing its embolization to the pulmonary or systemic vasculature [5,62,63]. When redundant, the CN can mimic an RA thrombus [64] or vegetations [44].

#### 3.2.3. Lipomatous Atrial Hypertrophy

Lipomatous atrial hypertrophy (LAH) is a benign cardiac lesion characterized by an excessive deposition of fat within the atrial septum. LAH typically involves the region of the interatrial septum, sparing the fossa ovalis with a pathognomonic dumbbell shape. The LAH prevalence is estimated to be approximately 5% in the general population (range 1–8%) [65,66]. It is more frequently detected among the elderly, females, and individuals with obesity [67]. LAH is generally an incidental finding on TTE [68]. However, some authors have described an increased prevalence of atrial arrhythmias in patients with LAH; moreover, in the case of severe LAH, heart failure symptoms secondary to the obstruction of RA filling have been consistently reported [69,70]. A symptomatic obstructive LAH should be surgically treated [71]. Fat deposition may involve not only the atrial septum but also the RA-free wall and/or the atrioventricular junction, simulating pathological RA masses. Even if TTE is the preferred screening method for LAH assessment, TEE may allow for a better visualization of smaller depositions of fat within the RA wall due to its superior spatial and temporal resolution. As demonstrated in the aforementioned clinical case 2, the areas of adipose infiltration within RA walls accidentally observed during echocardiographic examinations are generally characterized by a concordant motion with the wall of the RA, in synchrony with the phases of the cardiac cycle, as assessed by PW-TDI. Finally, a CT scan may provide incremental diagnostic information because of its high specificity in identifying fat accumulation detected as a homogenous hypodense mass with low density [72].

#### 3.2.4. Atrial Septal Aneurysm

Atrial septal aneurysm (ASA) is defined as the bulging of the interatrial septum into one or both atrial chambers >10 mm beyond the plane of the atrial septum, forming a saccular structure [73]. Its prevalence in the general population is estimated to be around 1–2.5% [74]. When protruding into the RA cavity (type 1R) [75], ASA can mimic RA tumors [76,77,78].

**Figure 4:**
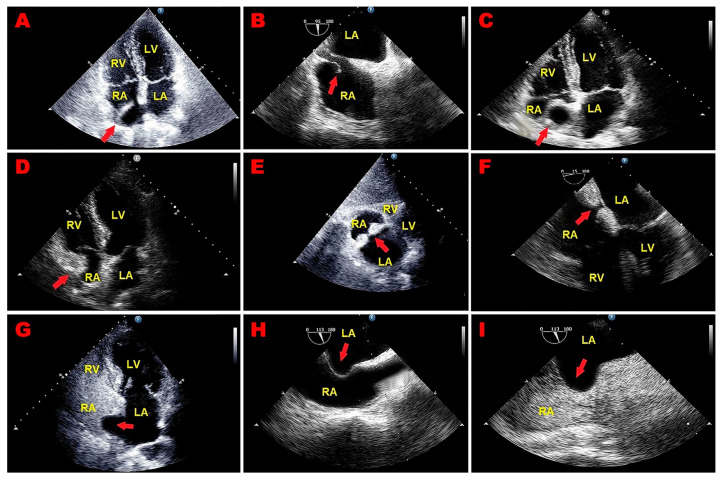
Representative examples of echocardiographically detected RA pseudomasses. (**A**) Transthoracic echocardiography. Apical four-chamber view, showing prominent Eustachian valve (red arrow). (**B**) Transesophageal echocardiography. Mid-esophageal bicaval view, revealing prominent Eustachian valve (red arrow). (**C**) Transthoracic echocardiography. Apical four-chamber view, revealing redundant Chiari’s network (red arrow). (**D**) Transthoracic echocardiography. Apical four-chamber view, highlighting lipomatous atrial hypertrophy involving RA free wall (red arrow). (**E**) Transthoracic echocardiography. Subcostal four-chamber view, demonstrating lipomatous atrial septal hypertrophy (red arrow). (**F**) Transesophageal echocardiography. Mid-esophageal four-chamber view, showing lipomatous atrial septal hypertrophy (red arrow). (**G**) Transthoracic contrast echocardiography. Apical four-chamber view, showing an atrial septal aneurysm (red arrow) protruding into the RA cavity, mimicking an RA mass, with no evidence of interatrial shunt on saline contrast echocardiography. (**H**) Transesophageal echocardiography. Mid-esophageal bicaval view, showing an atrial septal aneurysm (red arrow) protruding into the RA cavity, mimicking an RA mass. (**I**) Transesophageal contrast echocardiography. Mid-esophageal bicaval view, highlighting atrial septal aneurysm (red arrow) protruding into the RA cavity with no evidence of interatrial shunt on saline contrast echocardiography. LA, left atrium; LV, left ventricle; RA, right atrium; RV, right ventricle. (**A**–**C**,**E**–**I**) are reproduced from the paper [79].

Representative examples of echocardiographically detected RA pseudomasses are depicted in Figure 4A–I.

**Figure 5:**
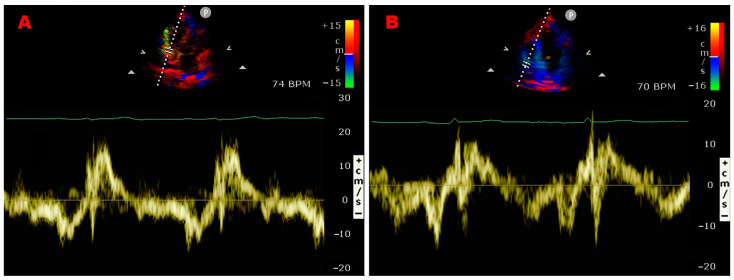
Examples of PW-TDI assessment of the systolic infolding of the lipomatous right atrioventricular junction (**A**) and of the RA infero-lateral wall (**B**). Spectral PW-TDI allows for the detection of a pattern of concordant motion in synchrony with the phases of the cardiac cycle, thus indicating RA pseudomasses. PW, pulsed wave; RA, right atrial; TDI, tissue Doppler imaging.

Figure 5 illustrates representative examples of PW-TDI assessment of RA pseudomasses.

### 3.3. Clinical Applications of PW-TDI

**Figure 6:**
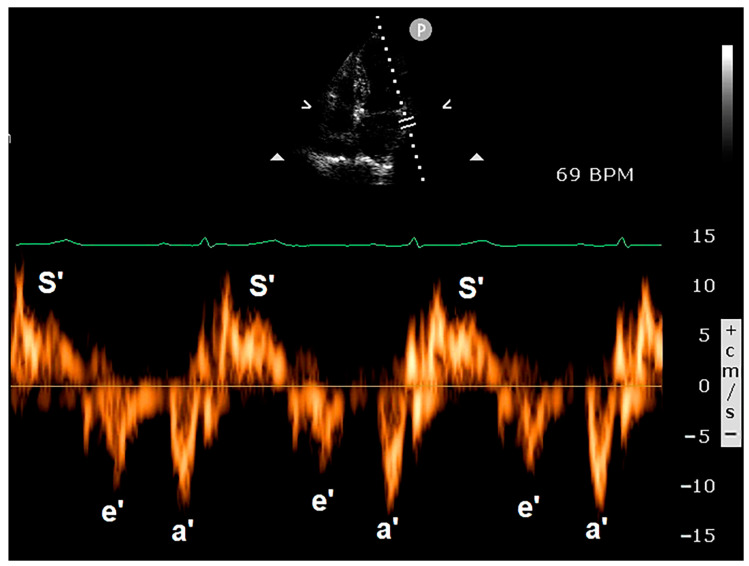
Spectral PW-TDI was obtained by placing the sample volume on the basal lateral wall of the left ventricle. The systolic wave (S’ velocity) represents myocardial contraction, while the two negative waves represent early diastolic myocardial relaxation (e’ velocity) and active atrial contraction in late diastole (a’), respectively. PW, pulsed wave; TDI, tissue Doppler imaging.

The use of PW-TDI for evaluating LV function was first reported by Isaaz et al. in 1989 [80]. The authors validated this innovative, non-invasive method for assessing the motion dynamics of the left ventricular (LV) posterior wall. In current clinical practice, PW-TDI is primarily used to quantify myocardial velocities throughout the cardiac cycle. For this purpose, the ultrasonographic system should be optimized to filter out the high-velocity signals of blood flow within the cardiac chambers and display only the low-velocity and high-amplitude signals of the wall motion velocities [81]. Regional quantification of myocardial velocities can be carried out at selected sites in either parasternal or apical windows. A small sample volume (usually 5 mm axial size) is used for accurate placement within the myocardium, whereas sampling cannot be localized to the endocardial or epicardial layers. Typically, in order to assess the regional systolic and diastolic myocardial velocities in the lateral and septal segments, a pulsed Doppler sampling is performed from the apical four-chamber view in the basal regions of the left ventricle adjacent to the mitral annulus [82]. Measurement of myocardial velocities from the apical views with PW-TDI reflects the LV longitudinal shortening and relaxation. On the other hand, velocities recorded in the parasternal views represent short-axis shortening and relaxation. Myocardial velocities recorded with PW-TDI have three main components over a cardiac cycle. These include a positive systolic wave (S’ velocity), which represents myocardial contraction, and two negative waves, which represent early diastolic myocardial relaxation (e’ velocity) and active atrial contraction in late diastole (a’), respectively (Figure 6).

S’ wave measures longitudinal LV contraction and is a surrogate of LV systolic function. S’ velocity was found to show good correlation with left ventricular ejection fraction (LVEF) [83]; S’ wave ≥7.5 cm/s is the accepted cut-off of normality [84]. S’ wave is related to the contraction of endocardial longitudinal fibers, which is largely responsible for long-axis function. The impairment in long-axis contraction commonly precedes changes in short-axis function. Several pathological conditions, such as hypertension, coronary artery disease, cardiomyopathies, and heart failure, have all been shown to alter subendocardial longitudinal function with a reduction in S’ velocity despite a preserved LVEF [85,86,87,88,89]. With regard to the early-diastolic velocities, these can be measured either from the septal or lateral annulus, but the current recommendation is that e’ velocity is expressed as the average of septal and lateral measurement (average e’) [82]. Generally, the e’ wave is higher in the lateral basal segments compared to the septal segments [90]. The e’ velocity correlates inversely with early diastolic pressure, thereby reflecting LV relaxation [91]. If the relaxation of the myocardium is abnormal, the e’ magnitude is decreased. Annular septal e’ >7 cm/s and annular lateral E’ >10 cm/s are considered within the range of normality [84]. PW-TDI is commonly used to assess LV filling pressures (LVFPs) and LV stiffness and is the preferred technique for routine clinical assessment of diastolic function [82]. The ratio of transmitral early filling velocity (E) to the early relaxation tissue velocity (e’), the so-called E/e’ ratio, strongly correlates with LVFPs [92]. A value  of <8 is considered a normal LVFP, whereas an average of septal and lateral E/e′  >14 is highly specific for a pulmonary capillary wedge pressure of >15 mmHg and, therefore, elevated LVFP [84,93]. An increased E/e’ ratio has been associated with adverse outcomes in various clinical settings [94,95,96].

Another clinical application of PW-TDI is the assessment of the systolic dyssynchrony between the septal and lateral walls, particularly in patients with severe systolic dysfunction and/or wide QRS due to complete left bundle branch block [97,98].

### 3.4. Implications for Clinical Practice

The use of PW-TDI for evaluating intracardiac masses has been used rarely in clinical practice. To date, only a few authors have made use of this technique for discriminating the fine movements of intracardiac masses. The pattern of incoherent motion detected by PW-TDI is typical of pathological cardiac masses due to the free oscillation of an anomalous structure with a motion direction and velocity independent and completely different compared to the surrounding tissue, with no correlation with the cardiac cycle. In this specific setting, PW-TDI can provide a detailed, accurate, and reproducible functional assessment of intracardiac pathological mass motility with potential prognostic implications; the higher the mass peak antegrade velocity, the higher the risk of embolic complications. Accordingly, this innovative echocardiographic parameter may be used for a more comprehensive prognostic risk stratification of patients with intra-cardiac pathological masses. Our study group demonstrated that a TDI-derived mass peak antegrade velocity ≥10 cm/s, as recorded at the level of the mobile portion of intraventricular thrombi, was independently associated with embolization and/or death at 1-year follow-up in a prospective cohort of patients with CAD history and apical thrombi [21]. Given that TTE implemented with PW-TDI can also be performed at the patient’s bedside, both the pattern of incoherent motion and an elevated mass peak antegrade velocity might also be easily collected in emergency scenarios, aiding clinicians in decision-making and treatment.

On the other hand, para-physiological structures, such as the aforementioned RA pseudomasses, are characterized by a pattern of concordant motion, with no difference in direction and phase compared with the surrounding tissue.

### 3.5. Limitations of PW-TDI

The main limitations of PW-TDI are the learning curve for the acquisition and analysis of TDI data, the variability in the ability to determine TDI velocities [99], a limited spatial resolution [90], and its angle dependency, i.e., the dependence on the angle between the ultrasound beam and the velocity directions (which means that if the angle of interrogation exceeds 20 degrees, the velocity may be underestimated) [100]. In fact, the Doppler technique depends on the parallel alignment of the Doppler beam to the moving objects, and incorrect angulations can disturb the result [101]. In addition, TDI is influenced by translational motion and tethering (normal apical segments pull an abnormal basal segment toward the apex). Additionally, one must bear in mind that single-point interrogation may not fully correspond to the real global myocardial mechanics [102].

## 4. Conclusions

The implementation of TTE with PW-TDI may facilitate the differential diagnosis between right-sided pathological and para-physiological cardiac masses.

The TDI-derived pattern of incoherent motion is typical of right-sided thrombi, myxomas, and vegetations, whereas right-sided pseudomasses are generally characterized by a pattern TDI of concordant motion synchronous with the cardiac cycle.

An increased TDI-derived mass peak antegrade velocity may represent an innovative marker of the embolic potential of mobile right-sided pathological masses.

In order to validate the clinical use of PW-TDI in the diagnostic and prognostic evaluation of patients with intra-cardiac masses, multicentric prospective studies are required.

## Data Availability

Data extracted from included studies will be publicly available on Zenodo (https://zenodo.org, accessed on 25 December 2024), pending acceptance by the journal.
